# A Rare Presentation of Hypereosinophilic Syndrome With Loffler’s Endomyocarditis, Encephalopathy, and Multiple Thromboembolic Strokes

**DOI:** 10.7759/cureus.46050

**Published:** 2023-09-27

**Authors:** Hassan Hussain, KVC Janaka, Naomali Amarasena, Sudeshan Senanayake, Dhanuka Sudam

**Affiliations:** 1 General Medicine, Sri Jayawardenapura General Hospital, Colombo, LKA; 2 Internal Medicine, Sri Jayawardenapura General Hospital, Colombo, LKA; 3 Cardiology, Sri Jayawardenapura General Hospital, Colombo, LKA; 4 Internal Medicine, University of Peradeniya, Kandy, LKA

**Keywords:** thromboembolism, platelet-derived growth factor receptor alpha, loffler's endomyocarditis, myocardial fibrosis, idiopathic hypereosinophilic syndrome

## Abstract

Idiopathic hypereosinophilic syndrome is a disorder with a high eosinophilic count for which no identifiable cause is evident. Herein we report a case of a 47-year-old male with a background history of hypereosinophilia, who presented with sudden onset altered level of consciousness and drowsiness for 1-day duration associated with a gradual onset progressive memory loss for 1-month duration. Based on clinical, biochemical, and imaging studies, a diagnosis of Loffler’s endomyocarditis was made for which he was treated with albendazole with diethylcarbamazine along with high-dose steroids. He made a successful recovery after 2 months of treatment.

## Introduction

Hypereosinophilic syndrome is a rare myeloproliferative disorder characterized by persistent high eosinophils associated with multiple organ damage [1]. There are many causes that are responsible for the development of this disease condition, hence idiopathic hypereosinophilic syndrome is diagnosed after excluding possible secondary and clonal causes of high eosinophilic count. The presence of a sustained absolute eosinophil count of >1500 cells per microliter in the peripheral blood for more than 6 months duration is mandatory for the diagnosis while no other identifiable cause for eosinophilia or signs of organ involvement should be evident [1,2]. Among the complications of hypereosinophilic syndrome cardiac complications like myocardial fibrosis and heart failure pose a significant threat to life.

## Case presentation

A 47-year-old male from a rural area in Sri Lanka, presented with sudden onset altered level of consciousness and drowsiness for 1-day duration associated with a gradual onset progressive memory loss for 1-month duration.

Prior to this admission, he had a history of gradual onset central burning type chest pain of moderate severity along with intermittent episodes of dyspeptic symptoms, loss of appetite, and mood changes for 1-month duration. He first sought medical advice for those symptoms 2 months back when he presented to the local hospital with the said symptoms. During that presentation, he had undergone basic investigations and was found to have an eosinophilic count of 58*10^3^/cumm^3^. The same findings were elicited in the blood picture and bone marrow biopsy as well. He was managed symptomatically and was discharged after a few days of hospital stay. His symptoms mildly improved over time and no hospital admissions or further follow-up studies were done, until this index admission.

With regards to the current presentation, apart from the said symptoms he complained of generalized body weakness, occasional dry cough, and dyspeptic symptoms but no history of fever, headache, photophobia, or any history of trauma to the head was noted. He denied any urinary symptoms, alteration of bowel habits, or joint pain, and no skin rashes or wounds were noted either.

He had no other medical comorbidities and his past surgical, drug, and allergic histories were unremarkable. He had no family history of hematological malignancies. He is a trishaw driver by profession and is a non-smoker and a teetotaler. He has no exposure to dust, or animal fur, and does not have pets at home.

Upon examination, he was drowsy and was not oriented in time, place, and person. His Glasgow Coma Scale (GCS) on admission was 13/15. He was an averagely-built male whose appearance seemed to be consistent with his chronological age. He was afebrile, and no skin rashes or generalized lymphadenopathy was noted. There was no conjunctival pallor or plethora, and no ankle edema was noted. His pulse rate was 100 beats per minute, with good volume and normal character. Peripheral pulses were present and there were no pulse deficits noted. He had a blood pressure of 130/80 mmHg. On cardiovascular system examination, the first and the second heart sounds were heard with no murmurs. His respiratory rate was 12 per minute with bilateral equal air entry. Vesicular breathing was present and lungs were clear. His abdomen was not distended and there was no organomegaly. His neurological examination did not reveal any focal neurological signs or signs of meningism. Fundus examination was normal. The rest of the systemic examinations were unremarkable.

In view of arriving at a diagnosis for the current clinical presentation, multiple investigations were performed (Table 1). 

**Table 1:** Investigations CSF: Cerebrospinal fluid; IgG: Immunoglobin G; IgE: Immunoglobin E; ANCA: Antineutrophil cytoplasmic antibody

Investigation		D1	D2	D4	D8	Normal range
Full blood count	Hemoglobin (g/dL)	11.1	11.1	13.7	14.4	11-13
	White cells (× 10^9^/L)	30.96	13.25	8.65	6.3	4.5-11
	Neutrophils (%)	12.2	36.9	52.2	39.4	50-70
	Lymphocytes (%)	13.3	25.5	29.8	43.5	20-40
	Eosinophils (%)	71.9	4.5	7.8	9.5	1-4
	Absolute eosinophilic count (× 10^9^/L)	22.26	0.60	0.67	0.60	<0.5
	Platelets (x 10^9^/L)	228	272	290	253	150-400
Serum electrolytes	Serum potassium (mmol/L)	3.8	-	-	3.8	3.5-5.5
	Serum sodium (mmol/L)	135	-	-	142.8	135-145
	Serum creatinine (µmol/L)	86	-	-	81	<100
Liver biochemistry	Aspartate transaminase (U/L)	36	-	-	-	<40
	Alanine transaminase (U/L)	36.39	-	-	-	<40
	Alkaline phosphatase (U/L)	83	-	-	-	<120
	Total bilirubin (mg/dL)	0.5	-	-	-	0.30-.20
Clotting profile	Prothrombin Time (sec)	11.2	10.9	12.3	16.7	11-13.5
	INR (international normalizing ratio)	0.83	0.8	0.91	2	<1
Infection screening	C-reactive protein (mg/L)	29	11	-	4	<5
	Erythrocyte sedimentation rate (mm in 1^st^ hour)	86	-	-	29	<20
	Urine full report	Normal				
	Urine culture	No growth				
	Retroviral studies	Negative				
	Stool full report and culture	Negative; no ova, cyst, or amoeba				
	Filarial antigens and antibodies	Negative				
	Toxocara canis IgG	Negative				
Other blood investigations	Creatine phosphokinase (µg/L)	12.5	-	-	-	10-120
	Blood urea (mg/dl)	39	-	-	-	15-40
CSF examination	Cell count	Normal	-	-	-	
	Protein	High	-	-	-	
Cardiac biomarkers	Troponin I (ng/ml)	2.64	-	-	-	<0.034
Immunoglobulin levels	IgE	Markedly elevated				
	IgG	Slightly elevated				
Imaging studies	Ultrasound scan of the abdomen (USS)	Normal study				
	Contrast-enhanced computed tomography (CECT) chest and abdomen	Normal study				
	Noncontrast computed tomography (NCCT) brain	Normal study				
	Magnetic resonance imaging (MRI) of the brain	Bilateral acute to subacute infarction involving frontal-parietal, occipital, and left cerebellum				
	2D echocardiography	Left ventricular thrombus measuring 2*6*1.4*4.4 cm with preserved ejection fraction				
	Cardiac MRI	Features of focal area of Loffler’s endomyocarditis in anterolateral wall of basal and mid ventricle with associated intracardiac thrombus formation				
	Upper gastrointestinal endoscopy (UGIE) and biopsy	No features of eosinophilic gastritis				
	Electroencephalogram (EEG)	Features of encephalopathy present				
	Carotid and vertebral artery Doppler	Normal				
	Venous Doppler	Normal; no thrombus detected				
Other investigations	Anti-nuclear antibody (ANA)	Not detected				
	Double-stranded DNA	Not detected				
	P-ANCA	Negative				
	C-ANCA	Negative				
	Serum vitamin B12	Above 97th percentile				
	Bone marrow aspiration and biopsy	Marked eosinophilia present				
	Blood picture	Marked eosinophilia present with moderate leucopenia and normochromic normocytic red blood cells; no primitive or abnormal cells present				

**Figure 1:**
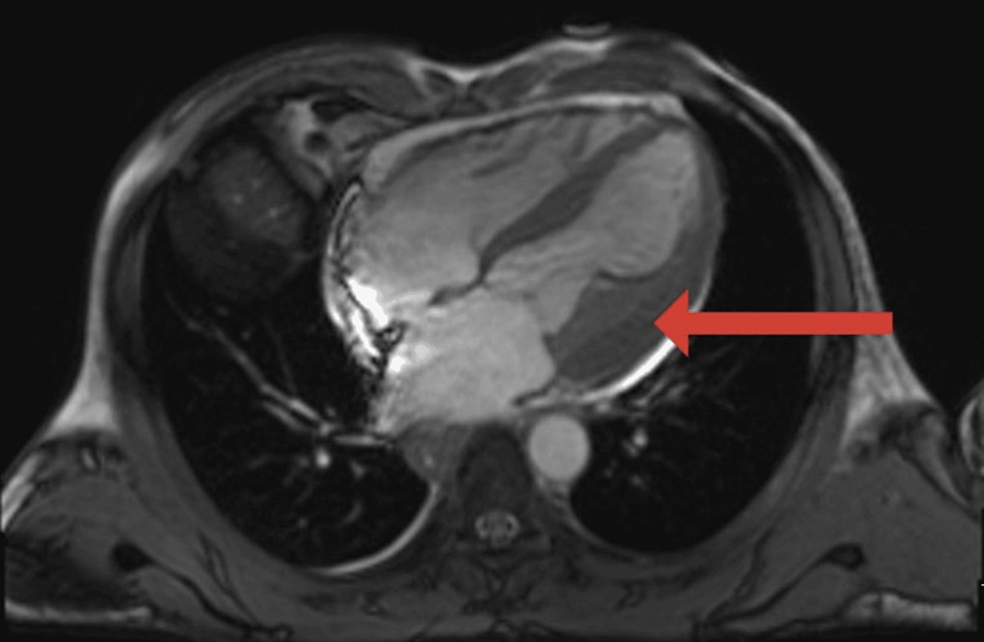
Cardiac MRI showing features of the focal area of Loffler’s endomyocarditis in the anterolateral wall of the basal and mid ventricle with associated intracardiac thrombus formation

Cardiac MRI showed features of the focal area of Loffler’s endomyocarditis in the anterolateral wall of the basal and mid ventricle with associated intracardiac thrombus formation (Figure 1).

**Figure 2:**
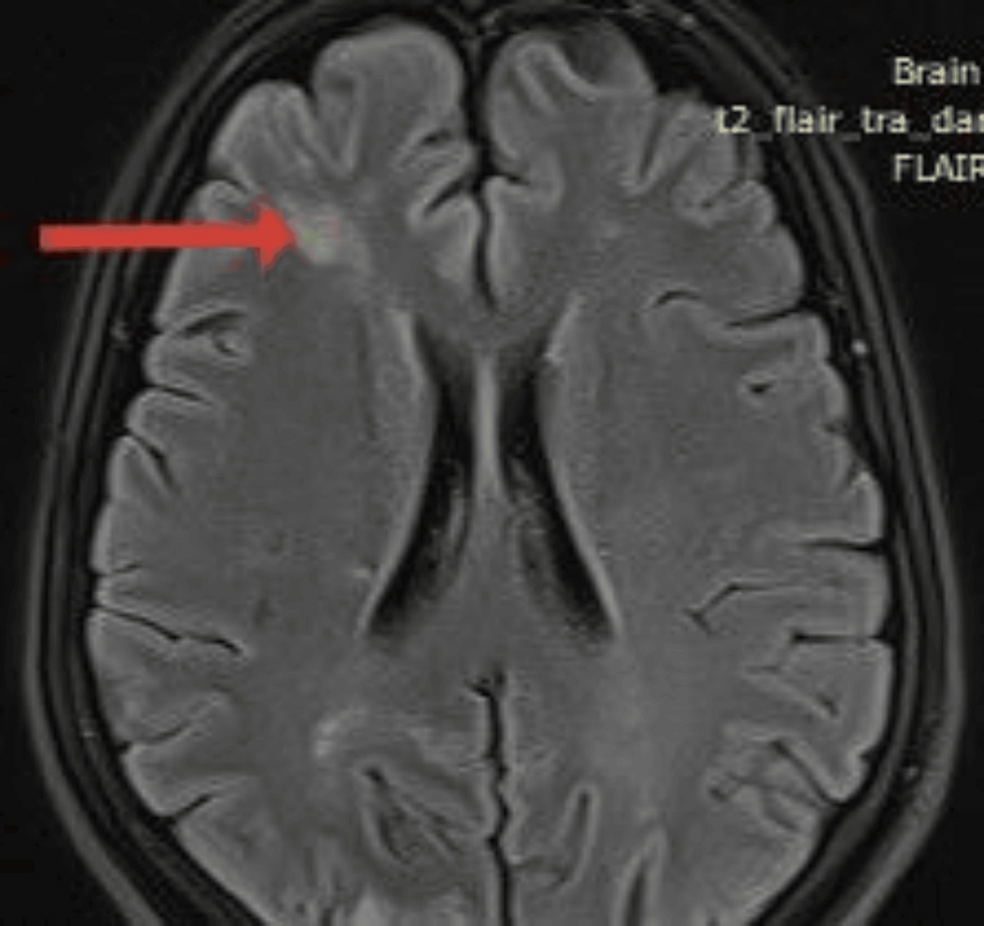
MRI of the brain showing bilateral acute to subacute infarction involving the frontal-parietal, occipital, and left cerebellum

MRI of the brain showed bilateral acute to subacute infarction involving the frontal-parietal, occipital, and left cerebellum (Figure 2). 

2D echocardiography showed a left ventricular thrombus measuring 2*6*1.4*4.4 cm with preserved ejection fraction (Video 1). 

**Video 1:** 2D echocardiography showing left ventricular thrombus

Based on the clinical and biochemical findings a diagnosis of idiopathic hypereosinophilic syndrome was made after excluding many secondary causes including infective, immunological, and connective tissue disorders. Unfortunately, clonal causes of hypereosinophilia could not be excluded as the patient was financially unstable to afford the studies. Upon diagnosis, he was initiated on albendazole with diethylcarbamazine along with oral prednisolone at 60 mg daily dose. He was also started on warfarin for anticoagulation.

Within 2 days of initiation of treatment, there was a significant reduction in eosinophilic count and a serum tryptase study was done which turned out to be within the normal range. Further evaluation with platelet-derived growth factor receptor alpha (PDGFRA) study was arranged in order to exclude inherited disorders associated with hypereosinophilia. It was found to be negative in this patient.

The patient was evaluated 2 months after initiation of steroids and a dramatic reduction in the previously noted cardiac thrombus was noted along with significant improvement in his clinical state.

## Discussion

Hypereosinophilia is caused by various pathophysiological mechanisms [3]. They can be broadly classified as primary and secondary based on the causative mechanisms. In the event that no causative factor is evident, it is classified as idiopathic. Primary causes of hypereosinophilia include neoplastic causes where there is clonal expansion of underlying stem cells or myeloid precursors. In secondary hypereosinophilia, there is overproduction of eosinophil poetic cytokines by other cell types and their polyclonal. This is classically associated with parasitic infection, certain solid tumors, and T-cell disorders [4]. Hypereosinophilic variants can be further divided into several clinically relevant subtypes namely myeloproliferative variants, T lymphocytic variants, familial idiopathic, organ-restricted hypereosinophilic conditions, and specific syndromes associated with hypereosinophilia [4]. The differential diagnoses that are usually considered are acute eosinophilic leukemia, chronic myeloid or myelomonocytic leukemia, and systemic macrocytosis with eosinophilia [1].

Eosinophilic overproduction occurs as a result of a primary molecular defect that involves hemopoietic cells or defects in signal transduction from the receptors that mediate eosinophilopoiesis. Considering the pathophysiology behind hypereosinophilic syndrome, there is eosinophil-mediated significant damage to the tissues that they infiltrate. The major target organs include the skin, lungs, gastrointestinal tract, and less commonly cardiovascular system and brain which are known to be life-threatening conditions if left untreated. Among the commonly involved organs skin plays a major role [5]. The skin manifestations include eczema, erythroderma, and generalized thickening of the skin, and when evaluated with biopsy it shows perivascular infiltrations without vasculitis. Mucosal involvement is extremely rare in hypereosinophilic syndrome. Pulmonary involvement results from eosinophilic infiltration of the lungs and subsequent fibrosis, heart failure, and pulmonary embolism [1]. The most common symptoms of pulmonary involvement include dyspnea, cough, and wheezing. Gastrointestinal involvement includes eosinophilic gastritis, enteritis or colitis causing weight loss, and non-specific abdominal pain [2]. Hepatic involvement may take the form of chronic active hepatitis, focal hepatic lesions, eosinophilic cholangitis, or Budd-Chiari syndrome. Even though these findings are commoner in association with hypereosinophilic syndrome none of these systemic manifestations were evident in this index case.

Cardiac involvement of this disease includes eosinophilic myocarditis which is a major cause of high mortality and morbidity associated with this condition. Clinical features of myocarditis include chest pain, exertional fatiguability, unexplained tachycardia, and arrhythmias. Lethal cardiac involvement is seen in PDGFRA-associated hypereosinophilic syndrome [3,4,6]. Eosinophil-mediated cardiac damage involves an increased number of eosinophils in conjunction with other ill-defined stimuli leading to extracellular deposit of eosinophilic granules proteins and evidence of eosinophilia is present at sites of myocardial injury. There are characteristic stages of myocardial injury; initially, the necrotic stage following the intermediate phase is characterized by thrombus formation along the damaged myocardium and finally evolves to the fibrotic stage which is characterized by altered cardiac function, heart failure due to restrictive cardiomyopathy or entrapment of chordae tendineae, leading to mitral or tricuspid regurgitation. An echocardiogram can be normal during the acute necrotic stage but contrast-enhanced cardiac MRI reliably detects all stages and aspects of eosinophilic-mediated heart damage including early stage of myocardial inflammation. Endomyocardial biopsy is reserved for rare cases when the diagnosis is uncertain.

Neurological disease may be complicated by thromboembolism, encephalopathy, and neuropathy. Cerebral-thromboembolism may manifest as stroke, or transient ischemic attack, with MRI features of multiple watershed infarcts. Encephalopathy can present with behavioral changes, confusion, ataxia, and memory loss

Once the hypereosinophilia is diagnosed, it is mandatory to perform investigations to find a cause for the hypereosinophilia. The initial investigations that are performed include liver function tests, creatine kinase, and troponin. Further evaluation is needed with imaging studies including electrocardiogram, echocardiogram, pulmonary function tests, chest radiographs, and contrast-enhanced computed tomography (CECT) abdomen, MRI brain as well as tissue biopsies whenever indicated. These investigations were performed in this patient and that led to the diagnosis of Loffler’s myoendocarditis. In addition, several biomarkers are under study for their predictive value of hypereosinophilic syndrome subtypes including serum tryptase, serum B12, immunoglobulin E (IgE), molecular testing for FIP1L1-PDGFRA mutation in peripheral blood, T lymphocyte phenotyping, and bone marrow biopsy [6].

The goal of treatment is to reduce absolute eosinophil count, amelioration of signs and symptoms, and prevention of disease progression [3]. The urgency of treatment depends on the severity of the disease. Indications for emergency treatment are significantly elevated absolute eosinophil count >100,000 cells/µL, signs and symptoms of pulmonary or neurological dysfunction in a setting of white blood cells >50x10^9, and signs and symptoms of potentially life-threatening complications of hypereosinophilia including acute heart failure, evidence of likely developing eosinophil mediated cardiac damage, thromboembolic phenomenon that can be present as diffuse watershed central nervous system infarction or focal central nervous system thrombo-emboli and pulmonary involvement [3].

High-dose glucocorticoids are preferred, 1 mg/kg of prednisolone, to 1 gram of methylprednisolone. It is critical to assess the patient’s risk of *Strongyloides* infection prior to administration of glucocorticoids since it can precipitate potentially fatal disseminated infection in infected patients [4]. Individuals with any potential exposure to *Strongyloides* should be treated empirically with ivermectin (200 mcg/kg/daily for 2 days). The expected response for high-dose steroids is 50% of the initial eosinophil count. Imatinib mesylate can be used in some steroid-refractory patients [4,6]. The other available medications are vincristine and hydroxyurea [4,6].

## Conclusions

Hypereosinophilic syndrome is a rare myeloproliferative disorder which has life threatening complications. Loffler's myoendocarditis is one such entity which needs to be looked into. As evident in our case, 2 months after initiation of steroids a dramatic reduction in the previously noted cardiac thrombus was noted along with significant improvement in the clinical state of this patient. Hence early identification and treatment of the disease and its complications is mandatory for a better outcome. 
